# The Gut Microbiome in Congenital Heart Disease: Dysbiosis, Intestinal Barrier Injury, and Translational Opportunities Across the Childhood—A Narrative Review

**DOI:** 10.3390/children13050668

**Published:** 2026-05-11

**Authors:** Alina-Costina Luca, Dana Elena Mindru, Solange Tamara Rosu, Cosmin Diaconescu, Eduard Vasile Rosu, Elena Țarcă, Heidrun Adumitrăchioaiei, Dana-Teodora Anton-Paduraru

**Affiliations:** 1 Department of Pediatrics, Faculty of Medicine, Grigore T. Popa University of Medicine and Pharmacy Iasi, RO-700115 Iasi, Romania; alina.luca@umfiasi.ro (A.-C.L.); diaconescu.cosmin@email.umfiasi.ro (C.D.); eduard.rosu@umfiasi.ro (E.V.R.); dana.anton@umfiasi.ro (D.-T.A.-P.); 2 Department of Nursing, Faculty of Medicine, Grigore T. Popa University of Medicine and Pharmacy Iasi, RO-700115 Iasi, Romania; 3 Department of Surgery II—Pediatric Surgery and Orthopedics, Faculty of Medicine, Grigore T. Popa University of Medicine and Pharmacy Iasi, RO-700115 Iasi, Romania; tarca.elena@umfiasi.ro; 4 Department of Pediatrics, Faculty of Medicine, George Emil Palade University of Medicine, Pharmacy, Science and Technology Targu Mureș, RO-540142 Targu Mures, Romania; heidrun.adumitrachioaiei@umfst.ro

**Keywords:** congenital heart disease, gut microbiome, cardiopulmonary bypass, intestinal barrier, necrotizing enterocolitis, Fontan, neurodevelopment

## Abstract

**Highlights:**

**What are the main findings?**

- Early infancy is a key period for gut microbiome and immune development, shaped by delivery mode and feeding.
- Congenital heart disease, common intensive care and perioperative exposures can predispose to gut microbiome imbalance and impaired intestinal barrier function.
- Cardiopulmonary bypass can act as a second hit, linking increased intestinal permeability and endotoxin in the bloodstream to outcomes such as necrotizing enterocolitis, feeding intolerance and infection-risk patterns.

**What are the implications of the main findings?**

- The gastrointestinal tract should be considered a vulnerable target organ in congenital heart disease from neonatal to chronic stages.
- Nutrition strategies, including human milk exposure, are practical targets, while microbiome-directed supplements require explicit safety considerations.
- Future studies should combine microbiome profiling with measures of intestinal barrier injury, inflammation and tissue perfusion in longitudinal cohorts and intervention trials.

**Abstract:**

Congenital heart disease (CHD) is the most common congenital anomaly worldwide and is associated with substantial infant morbidity and mortality. This narrative review synthesizes evidence linking CHD to alterations in the gut microbiome across neonatal, perioperative, and chronic stages and highlights a gut–heart–immune framework in which microbial imbalance, intestinal barrier dysfunction, and systemic inflammation may interact to influence clinical outcomes. Early infancy represents a potential window for microbiome and immune development, shaped by delivery mode and feeding, with many breastfed infants developing a *Bifidobacterium*-dominant community supported by human milk oligosaccharides. In CHD, abnormal splanchnic perfusion and hypoxemia, together with intensive care and perioperative exposures (fasting, delayed enteral feeding, antibiotics, acid suppression), may predispose to dysbiosis and impaired barrier function. Cardiac surgery with cardiopulmonary bypass can act as a “second hit,” with evidence of increased gut permeability, endotoxemia, inflammatory activation, and biomarker signals of enterocyte injury and tight-junction disruption. Clinically, these mechanisms align with gut-sensitive outcomes including necrotizing enterocolitis (especially in ductal-dependent lesions), feeding intolerance, and postoperative infection-risk phenotypes. Interventions show mixed evidence: human milk exposure appears protective for NEC risk, synbiotics demonstrated outcome benefits in a randomized trial of cyanotic CHD infants, while probiotics may modify dysbiosis without consistently preventing intestinal injury and require careful safety frameworks. Key research gaps include the need for longitudinal stage-based cohorts, integration of microbiome profiling with barrier injury and perfusion markers, and standardized safety monitoring in intervention trials.

## 1. Introduction

Congenital heart disease (CHD) refers to structural abnormalities of the heart that develop before birth. About 1 in 100 children is born with a congenital heart defect, sometimes associated with genetic or chromosomal abnormalities such as Down syndrome. Risk factors include heavy alcohol use during pregnancy, exposure to certain medications, and maternal viral infections in the first trimester. In addition, the risk is higher if a parent or sibling has congenital heart disease. These defects may involve abnormal heart valves, atrial or ventricular septal defects, stenosis, or heart muscle abnormalities, which can disrupt blood flow and lead to complications such as heart failure and, in severe cases, death. Early symptoms can be variable and subtle, but may include shortness of breath, reduced exercise tolerance, fatigue, or a heart murmur detected on examination. Diagnosis is typically established with echocardiography, supported by tests such as electrocardiography, chest X-ray, cardiac MRI, and cardiac catheterization, while treatment depends on severity and may range from medications for symptom control to catheter-based procedures or surgery, and rarely heart transplantation for end-stage disease [1].

CHD is the most common congenital anomaly worldwide, with a birth prevalence commonly cited around 10 to 12 per 1000 live births, and it remains a major contributor to infant morbidity and mortality [2,3]. These epidemiologic facts highlight a growing population of infants and children exposed to prolonged hospitalization, repeated antibiotic courses, and complex perioperative trajectories, and they shift the clinical focus from survival alone toward potentially modifiable drivers of morbidity such as infection, feeding intolerance, necrotizing enterocolitis (NEC), impaired growth, and longer-term systemic inflammatory phenotypes [4].

Beyond surgical repair and hemodynamic optimization, the gastrointestinal tract is increasingly recognized as an important factor in pediatric CHD care [4]. The human gut harbors a dense and diverse microbial ecosystem that contributes to host well-being and these microbes interact with the host immune system in ways that can influence disease phenotypes [5]. Early infancy represents a critical window in which gut microbial colonization and immune maturation develop, and microbial exposure is proposed to be an important driver of immune development during this period [6]. Disruption of intestinal barrier integrity can permit translocation of microbial products and amplify systemic inflammation, providing a biologically plausible route by which gut perturbations might affect extra-intestinal organs [7].

Infants with critical CHD experience multiple exposures that can reshape microbiome assembly, including altered gut perfusion and oxygen delivery, perioperative fasting and delayed enteral feeding, frequent antibiotic administration and intensive care–related environmental pressures [8,9].

This “gut vulnerability” matters clinically because CHD infants are at risk for intestinal complications such as necrotizing enterocolitis (NEC), particularly in ductal-dependent lesions where systemic perfusion patterns may be precarious [10,11].

Importantly, the microbiome signal may extend beyond the immediate perioperative window, because patients with Fontan circulation have been reported to exhibit gut dysbiosis linked to hemodynamic compromise and systemic inflammation [12]. Dysbiosis has also been described in Fontan patients with protein-losing enteropathy, reinforcing the hypothesis that intestinal congestion and lymphatic dysfunction may interact with gut ecology [13]. In early childhood CHD cohorts, gut microbiome differences have been associated with growth parameters and prospective work has begun to explore links between early-life microbiome patterns and neurodevelopmental outcomes [14,15]. Together, these data support a “gut–heart–immune” framework in which microbial composition, barrier integrity, and inflammation may influence both short-term complications and longer-term CHD phenotypes [4].

The goal of this narrative review is to synthesize current evidence connecting the gut microbiome with congenital heart disease across the neonatal, perioperative, and chronic stages, to summarize plausible mechanistic pathways (including dysbiosis, epithelial barrier dysfunction and systemic inflammation) that may link gut perturbations to clinical outcomes, and to identify translational opportunities where microbiome and intestinal barrier findings may inform risk stratification, perioperative monitoring, nutrition strategies, microbiome-directed interventions, and the design of future longitudinal and interventional studies.

## 2. Normal Early-Life Microbiome Development

Gut microbial colonization accelerates around birth and is shaped by maternal and environmental exposures, with delivery mode influencing the initial microbiota across neonatal body sites [16]. During the first days to weeks, early communities are often enriched in facultative anaerobes, enabling a transition toward strict anaerobes as the intestinal environment becomes more reduced [17]. In many healthy, breastfed infants, the gut becomes *Bifidobacterium*-dominant, reflecting strong diet–microbe selection pressures in early life [18].

Human milk contains human milk oligosaccharides (HMOs) that infants largely do not digest directly, but which act as selective substrates that promote bifidobacterial growth and cooperative networks within the infant gut ecosystem [19]. Breastmilk-associated bifidobacteria can generate bioactive metabolites (e.g., aromatic lactic acids) that are linked to immune-relevant signaling pathways, supporting a mechanistic basis for microbiome–immune crosstalk in infancy [20]. More broadly, early microbial exposure is viewed as a “window of opportunity” for immune education, with persistent consequences when host–microbe interactions are perturbed [21].

Longitudinal cohort data show that the infant gut microbiome changes rapidly in composition and function across the first year, with major influences from mode of delivery and feeding. Importantly, maturation toward a more “adult-like” configuration is strongly associated with weaning or cessation of breastfeeding, rather than solid food introduction alone [18]. Across early childhood, the gut microbiome follows reproducible developmental trajectories with identifiable phases and becomes progressively more stable by approximately 3–4 years of age [22,23]. These developmental features, particularly the transition toward strict anaerobes, the establishment of a *Bifidobacterium*-dominant community, and human milk oligosaccharide-driven colonization, represent potential points of vulnerability in infants with congenital heart disease, as discussed in the following section.

## 3. The Role of CHD in Altering the Microbiome

Children with CHD may be biologically and iatrogenically predisposed to gut dysbiosis because cardiovascular physiology directly shapes splanchnic perfusion and oxygen delivery. In cyanotic and low cardiac output states, abnormal gut perfusion and hypoxemia can stress the intestinal epithelium and may interfere with normal microbial colonization trajectories [24]. Consistent with this physiology-first concept, pediatric data show that patients with congenital heart defects have abnormal gut permeability compared with healthy children, even before considering operative factors [25]. The available evidence should also be interpreted according to clinical stage. Neonatal critical congenital heart disease cohorts mainly inform the concept of early dysbiosis and preoperative vulnerability, perioperative and postoperative cohorts support the role of cardiopulmonary bypass and barrier injury as a second hit, while Fontan cohorts represent a distinct chronic physiology in which congestion-related dysbiosis and inflammation may persist beyond the surgical period [8,9,12,13,26]. Fontan-related intestinal complications are discussed separately in the clinical consequences section.

In parallel, early-life CHD cohorts demonstrate that dysbiosis can be present very early in life, with reported depletion of typical infant commensals and enrichment of opportunistic taxa in critical CHD neonates [9]. A major amplifier is cardiac surgery with cardiopulmonary bypass (CPB), which acts as a “second hit” through ischemia–reperfusion injury, systemic inflammation, and barrier disruption [25]. Children undergoing surgery for CHD have been shown to be at increased risk of intestinal mucosal injury and endotoxemia with endotoxin activity being correlated with postoperative outcome variables [26]. Translational profiling further supports that CHD patients may have baseline microbiome differences that are exacerbated following surgery with CPB, alongside postoperative signals of intestinal barrier dysfunction [8]. Mechanistic work in a piglet CPB model similarly demonstrates rapid post-CPB shifts in the microbiome, barrier dysfunction, and inflammatory/metabolic perturbations, providing additional mechanistic plausibility for a gut-derived inflammatory contribution after bypass [27].

Beyond physiology and CPB, Intensive Care Unit (ICU) and perioperative exposures common in CHD care strongly influence microbiome assembly during the most plastic developmental window [24]. Broad-spectrum antibiotics, frequently administered in neonatal and cardiac ICUs, can cause marked shifts in infant gut microbiome composition and select for antimicrobial resistance features when given during early assembly phase of the neonatal microbiome [28]. Acid-suppressive therapy is another relevant modifier, as proton pump inhibitor exposure has been associated with significant alterations in gut microbiota composition in large human cohorts [29]. Feeding interruptions (perioperative fasting, delayed enteral feeding) and reduced human milk exposure may further skew early colonization. Conversely, preoperative exclusive human milk feeding in complex CHD has been associated with lower NEC risk, supporting nutrition as a clinically relevant microbiome-facing lever in this population [30].

Finally, in later CHD physiology, chronic congestion, particularly in Fontan circulation, may sustain dysbiosis and systemic inflammation long after the perioperative period [12,13].

## 4. Perioperative Gut Injury and Barrier Dysfunction

Cardiac surgery with cardiopulmonary bypass (CPB) can precipitate gastrointestinal injury through a coupled “inflammation–ischemia–endotoxemia” cascade. Contact of blood with the artificial circuit activates innate immune and coagulation pathways, while a later component is driven by ischemia–reperfusion and endotoxin-related amplification of systemic inflammation [31,32].

At the gut level, non-physiologic CPB conditions can impair mucosal transport and increase permeability, consistent with hypoperfusion and barrier stress during bypass [33]. In humans undergoing CPB, dual-sugar absorption testing demonstrates a marked postoperative rise in gut permeability. Mucosal tonometry data also linked greater permeability with evidence of splanchnic acidosis in a subset of patients [34]. A randomized study in cardiac surgery patients further supports CPB-associated increases in gastrointestinal permeability and suggests permeability may be modulated by perioperative vasoactive strategy (dopamine vs. dopexamine), even if the precise mechanism remains uncertain [35]. Permeability changes can also coexist with circulating endotoxin and postoperative systemic responses in cardiac surgery cohorts, reinforcing the plausibility of a gut-derived inflammatory contribution [36]. In neonates after cardiac surgery, intestinal permeability may remain elevated into the second postoperative week, indicating that barrier dysfunction can persist beyond the immediate ICU window [37].

In CHD specifically, pediatric studies document perioperative endotoxemia and inflammatory mediator release around surgery. Preoperative endotoxemia is common in children with CHD and endotoxin-related markers rise further after CPB alongside increases in inflammatory cytokines such as IL-6. A clinically meaningful subset of patients also develops significant postoperative hemodynamic disturbance [38]. During pediatric cardiac surgery, circulating endotoxin and tumor necrosis factor (TNF) can increase during and after bypass, supporting a mechanistic link between CPB and inflammatory activation [39]. In pediatric cohorts, TNF and IL-8 have been reported to correlate with bypass duration and to associate with systemic inflammatory response and multiple organ dysfunction phenotypes, even when endotoxin itself does not strictly track bypass time [40]. Additional work supports the concept of endogenous endotoxemia of intestinal origin during CPB and suggests that perfusion characteristics (flow type) may influence this signal [41]. More directly, children undergoing surgery for CHD demonstrate measurable intestinal injury and endotoxemia during the perioperative course, linking gut damage to systemic inflammatory biology in this setting [26].

Biomarker studies strengthen the mechanistic chain from enterocyte injury to tight-junction disruption to microbial product translocation. Intestinal fatty acid-binding protein (iFABP), an enterocyte cytosolic protein released with epithelial injury, rises in higher-risk CPB patients and has been associated with subsequent gastrointestinal complications [42]. In pediatric cardiac surgery, iFABP and claudin-3 (a tight-junction associated marker) increase after extracorporeal circulation. Furthermore, their dynamics track postoperative inflammatory mediator patterns, consistent with barrier injury biology during and after bypass [43]. More recent surgical cohorts also support iFABP as a clinically relevant signal for gastrointestinal injury in the setting of cardiac surgery [44]. Clinically, postoperative intestinal epithelial barrier dysfunction in children with CHD has been linked to identifiable perioperative characteristics, providing a framework to phenotype “barrier-risk” trajectories [45].

Endotoxin activity assays provide complementary evidence that barrier injury can translate into systemic endotoxin burden in pediatric bypass populations. In an exploratory pediatric cohort, endotoxin activity commonly exceeded reference thresholds post-CPB. In addition, higher endotoxin activity was associated with CPB duration and postoperative severity markers (including lactate, vasoactive requirements, ventilation duration) [46]. In neonates undergoing cardiac surgery, endotoxin activity has similarly been quantified perioperatively, supporting the relevance of endotoxin biology in the most vulnerable age group [47].

Experimental models provide mechanistic corroboration at the tissue level. In a rat CPB model, tight-junction architecture is disrupted (including reductions in occludin and ZO-1) alongside increases in biochemical markers consistent with barrier injury and permeability shifts, supporting biological plausibility for CPB-driven epithelial damage [48]. Finally, perioperative host detoxification capacity may modify the endotoxin signal. In infant cardiothoracic surgery cohorts, alkaline phosphatase activity trajectories correlate with postoperative support and inflammation, aligning with the concept that perioperative variation in endotoxin-handling pathways could influence systemic inflammatory phenotypes [49,50].

Taken together, these findings support the gut–heart–immune framework by showing that perioperative epithelial barrier injury and endotoxemia may represent key mechanisms through which cardiac surgery amplifies systemic inflammation and contributes to gut-sensitive outcomes in children with congenital heart disease.

## 5. Clinical Consequences: Necrotizing Enterocolitis, Feeding Intolerance and Infection-Risk Phenotypes

### 5.1. Necrotizing Enterocolitis (NEC)

Infants with CHD are at increased risk of NEC which carries substantial morbidity and mortality rate, including term infants in whom CHD is a commonly implicated risk factor [11].

In neonates with CHD, NEC risk factors and outcomes have been characterized in large pediatric cohorts, with lesion complexity (e.g., hypoplastic left heart syndrome) and prematurity among important associated factors [51].

Ductal-dependent CHD represents a particularly vulnerable subgroup. Studies in this population have evaluated the relationship between enteral feeding and NEC risk in the setting of prostaglandin dependence and altered systemic perfusion [10]. Hemodynamic mechanisms are supported by Doppler evidence that persistent diastolic flow reversal in the abdominal aorta is associated with increased NEC risk in term infants with CHD, consistent with an intestinal hypoperfusion phenotype related to “diastolic steal” [52].

Nutritional exposures may modify risk: preoperative exclusive human milk feeding has been associated with lower preoperative NEC risk in neonates with complex CHD, whereas higher preoperative feeding volumes and cow’s-milk formula exposure have been linked to increased NEC risk in that cohort [30].

Postoperatively, early enterocyte injury measured by circulating iFABP has been associated with subsequent NEC after infant cardiothoracic surgery, supporting objective biomarker-based risk stratification [53].

At the practice level, a systematic review and meta-analysis found no overall association between preoperative feeding status and NEC in neonates awaiting cardiac surgery, highlighting ongoing uncertainty and the need for phenotype-stratified feeding strategies [54].

These findings suggest that feeding strategies in CHD should not be uniform across lesion types. Rather, infants with ductal-dependent lesions and impaired systemic perfusion may require more individualized approaches, whereas the lack of a consistent overall association between preoperative feeding status and necrotizing enterocolitis highlights why such stratification is not yet standardized in practice [10,30,52,54].

### 5.2. Feeding Intolerance and Feeding Difficulties

Feeding intolerance (FI) and feeding difficulties are common in pediatric CHD care and can delay achievement of full enteral nutrition and contribute to longer hospitalizations and worse outcomes [55].

In neonates undergoing cardiac surgery, a substantial proportion require tube feeding at discharge. Identifiable clinical factors (including genetic syndrome and palliative procedure before biventricular repair) are associated with prolonged feeding-tube use [56].

Mechanistically, prospective perioperative studies demonstrate that postoperative FI can be associated with dysbiosis, elevations in circulating epithelial barrier dysfunction markers (e.g., claudin-2, claudin-3, FABP2), and depletion of stool and plasma short-chain fatty acids (SCFAs), linking the intestinal milieu to postoperative feeding outcomes [57].

Because perfusion may modulate tolerance, postoperative monitoring of splanchnic regional oxygenation during feeds has been shown to be feasible in neonates recovering from CHD surgery, supporting physiologic monitoring as a candidate tool to guide feeding advancement and potentially reduce gut complications [58].

In infants after congenital heart surgery, retrospective data suggest that achieving predefined enteral nutrition targets during the acute postoperative phase may be associated with improved clinical outcomes without necessarily increasing gastrointestinal complications, although prospective validation is required [59]. Interventional nutrition strategies also show promising signals. Early high-energy feeding protocols after congenital heart surgery have been associated with improved growth and reduced cardiac ICU stay, with reported reductions in ventilator time and postoperative infection rates in that study setting [60].

### 5.3. Infection-Risk Phenotypes

Postoperative infection is a major contributor to morbidity and resource utilization after pediatric cardiac surgery. Large multicenter datasets have enabled development of risk estimation models for major infections (e.g., septicemia, mediastinitis, endocarditis) and quantification of their impact on mortality and length of stay [61].

In congenital heart surgery programs in resource-limited settings, postoperative infections have been linked to higher mortality and longer ventilation and ICU stays, emphasizing the importance of prevention and quality-improvement efforts [62].

In children with CHD receiving inpatient cardiac care, laboratory-confirmed bloodstream infections are frequent and commonly involve Gram-positive organisms (including Enterococcus faecalis and Staphylococcus epidermidis) [63]. This pattern may be interpreted in light of the perioperative barrier dysfunction described earlier, where increased intestinal permeability, endotoxemia and epithelial injury support the plausibility of microbial product translocation across a compromised gut barrier [26,45,46]. Within this framework, the bloodstream infection phenotypes observed in children with congenital heart disease may reflect not only device- and hospital-related risk, but also the contribution of a stressed intestinal barrier to systemic infectious vulnerability [63,64]. Authors have proposed that bacterial translocation through injured gut mucosa may contribute even when formal mucosal barrier injury definitions are not met [63].

A mechanistic link between CPB physiology and infection susceptibility is supported by prospective adult cardiac surgery data showing that endotoxemia at the conclusion of CPB is associated with increased risk of postoperative infections, reinforcing the plausibility of gut-derived inflammatory signaling as a risk amplifier [64].

Microbiome-directed interventions may influence infection risk. In a randomized trial of infants with cyanotic CHD, enteral synbiotics were associated with reduced nosocomial sepsis, NEC, and death compared with placebo [65].

Safety must be addressed explicitly in high-risk postoperative cardiac populations, as probiotic-associated bacteremia has been reported after pediatric cardiac surgery, underscoring the need for careful patient selection, product quality assurance, and infection-control measures [66].

### 5.4. Fontan Circulation and Protein-Losing Enteropathy

Fontan physiology appears to represent a distinct chronic gut-related phenotype within congenital heart disease. In this setting, gut dysbiosis has been linked to hemodynamic compromise and systemic inflammation, suggesting that microbiome alterations are not limited to the perioperative period but may persist in later disease stages [12]. In addition, dysbiosis has been described in Fontan patients with protein-losing enteropathy, supporting the concept that chronic intestinal congestion and altered gut ecology may interact in this complication [13]. Together, these observations indicate that Fontan circulation should be considered separately from perioperative CHD states, as it reflects a chronic hemodynamic environment in which gut dysfunction may contribute to ongoing inflammation and intestinal morbidity [12,13].

## 6. Microbiome-Targeted Interventions

Microbiome-targeted strategies in CHD currently fall into four practical categories: (i) nutrition-first approaches (human milk, feeding protocols), (ii) biotic supplementation (probiotics, synbiotics), (iii) iatrogenic dysbiosis mitigation (antibiotic stewardship, minimizing unnecessary acid suppression), and emerging metabolite-directed or ecosystem replacement approaches (e.g., bile-acid modulation in Fontan, fecal microbiota transplantation).

To provide a concise overview of the intervention-related evidence discussed in this section, Table 1 summarizes the main studies evaluating nutritional strategies, probiotic or synbiotic approaches, safety signals, and emerging metabolite-directed interventions in the congenital heart disease gut–microbiome axis.

### 6.1. Nutritional Approaches

Preoperative exclusive human milk feeding has been associated with a lower risk of preoperative NEC in neonates with complex CHD, supporting human milk as a pragmatic, microbiome-facing intervention in high-risk lesions [30].

However, feeding practices remain controversial. A systematic review and meta-analysis found no overall association between preoperative feeding status and NEC in infants awaiting CHD surgery, underscoring heterogeneity by lesion type, perfusion risk, and institutional practice [54].

Most nutrition data in CHD are nonrandomized, sensitive to confounding (lesion severity, prostaglandin exposure, antibiotic duration, timing to surgery) and rarely paired with mechanistic microbiome endpoints [11,54].

### 6.2. Probiotics and Synbiotics

A blinded randomized controlled trial in cyanotic CHD infants reported that enteral synbiotics were associated with improved outcomes (including reductions in nosocomial sepsis, NEC and death signals in that cohort), suggesting potential benefit in selected high-risk phenotypes [65].

In contrast, a randomized pediatric cardiac surgery study found that probiotics corrected postoperative dysbiosis after CPB but did not sufficiently reduce CPB-associated intestinal injury endpoints, highlighting that “microbiome normalization” does not automatically translate into mucosal protection in the acute perioperative phase [67].

A CHD-specific retrospective cohort in ductal-dependent CHD suggested possible NEC reduction in a subgroup (e.g., aortic arch malformations) with dual-strain probiotics, but the overall cohort effect was not statistically definitive, illustrating the sample-size challenge for NEC endpoints [68].

Cross-study interpretation remains difficult because the available probiotic and synbiotic studies differ in formulation type, strain selection, dosing, duration of administration, and timing relative to surgery, which likely contributes to the inconsistency of reported effects across CHD populations [65,67,68].

### 6.3. Safety and Patient Selection

Probiotic-associated invasive infections are uncommon but documented in pediatrics. A systematic review identified multiple pediatric cases of invasive infection linked to probiotic organisms, supporting caution in high-risk hosts [70].

In postoperative pediatric cardiac surgery populations, probiotic-associated bacteremia has been reported, including concerns about possible cross-contamination in ward settings, reinforcing the need for clear contraindications (e.g., profound immunocompromise, unstable critical illness), strict line care and product handling protocols when probiotics are used [66].

On the other side, many interventional reports do not standardize surveillance for probiotic bloodstream infection or specify microbiology workflows to detect probiotic strains, which complicates true risk estimation [66,71].

### 6.4. Iatrogenic Dysbiosis Mitigation

Early-life exposure to broad-spectrum antibiotics can produce large shifts in infant microbiome development and select for antimicrobial resistance features, making antibiotic stewardship and de-escalation biologically compelling in CHD pathways where antibiotic exposure is common [28].

Proton pump inhibitor exposure has been associated with significant alterations in gut microbiota composition in large human cohorts, supporting minimization of unnecessary acid suppression when clinically safe [29].

### 6.5. Emerging and Experimental Directions

In Fontan physiology, dysbiosis and systemic inflammation have been linked to failed hemodynamics, motivating exploration of metabolite-directed interventions rather than community composition alone [12].

Bile-acid modulation is one example: a prospective randomized crossover pilot trial design has been published evaluating colesevelam to reduce bile acids in adult Fontan patients [69].

Another experimental direction is fecal microbiota transplantation (FMT). This represents a powerful ecosystem-replacement strategy with pediatric experience mainly in recurrent C. difficile infection and inflammatory bowel disease contexts, but pediatric reviews emphasize special safety considerations given the developing microbiome, not to mention that there is no established CHD perioperative role at present [72].

These approaches are not yet supported by robust CHD-specific efficacy data. They require careful donor or product screening, mechanistic endpoints and CHD-appropriate safety frameworks [71,72].

## 7. Discussion

The available evidence supports a staged gut–heart–immune model in congenital heart disease, but the strength of evidence is not uniform across all components of this framework. The most consistent data concern perioperative barrier injury, endotoxemia, and inflammatory activation, particularly around cardiopulmonary bypass, where human and experimental studies converge in supporting a “second hit” mechanism. By contrast, the evidence for broader clinical consequences such as feeding intolerance, infection-risk phenotypes, growth, and neurodevelopment remains more heterogeneous and is often associative rather than causal. This distinction is important, because it suggests that the gut may function both as a direct contributor to morbidity in some settings and as a marker of physiologic stress in others.

A mechanistic interpretation of the reviewed literature suggests that the relative contribution of different drivers may vary across congenital heart disease phenotypes. In neonatal and ductal-dependent lesions, abnormal perfusion and hypoxemia appear especially relevant, helping explain why necrotizing enterocolitis risk is concentrated in hemodynamically vulnerable subgroups and why Doppler markers of impaired mesenteric perfusion are clinically informative. In the perioperative setting, cardiopulmonary bypass, epithelial injury, and endotoxemia appear to amplify this baseline vulnerability. At the same time, intensive care exposures such as antibiotics, acid suppression, fasting, and delayed enteral feeding are likely important modifiers of microbiome composition and barrier recovery, although their relative weight compared with hemodynamic factors remains uncertain. This is a key point where interpretation should remain cautious: the current evidence supports interaction between these factors but does not yet allow confident ranking of their individual contributions across all congenital heart disease populations.

The intervention literature further illustrates this complexity. Human milk exposure appears to be the most practical and biologically plausible microbiome-facing strategy, especially in relation to necrotizing enterocolitis risk. In contrast, probiotic and synbiotic studies show mixed results: benefit signals are reported in some high-risk groups, whereas perioperative correction of dysbiosis has not consistently translated into protection from intestinal injury. This discrepancy suggests that microbiome composition alone may not fully capture the biologic processes driving postoperative morbidity, and that barrier integrity, perfusion status, and systemic inflammatory context may be equally important determinants of outcome. It also reinforces the need to avoid overinterpreting microbiome modification as a surrogate for clinical benefit.

**Figure 1:**
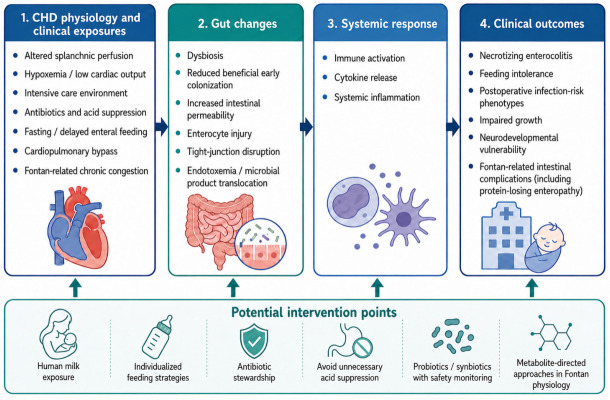
Conceptual gut–heart–immune pathway. Congenital heart disease physiology and perioperative exposures may promote gut dysbiosis, intestinal barrier injury, endotoxemia, and systemic inflammation, contributing to necrotizing enterocolitis, feeding intolerance, infection-risk patterns, impaired growth, neurodevelopmental vulnerability, and Fontan-related intestinal complications. Potential intervention points include human milk exposure, individualized feeding, antibiotic stewardship, cautious use of probiotics or synbiotics, and metabolite-directed approaches.

The proposed conceptual gut–heart–immune pathway linking congenital heart disease physiology, perioperative exposures, intestinal barrier dysfunction, systemic inflammation, and clinical outcomes is summarized in Figure 1.

An additional area that deserves greater attention is neurodevelopment. Early-life microbiome patterns have already been associated with both growth and neurodevelopmental outcomes in infants with congenital heart disease, suggesting that gut dysbiosis may extend beyond perioperative and gastrointestinal morbidity into longer-term developmental trajectories. However, the current evidence remains limited and should be interpreted cautiously. These studies are best viewed as hypothesis-generating rather than definitive, particularly because neurodevelopment in congenital heart disease is shaped by multiple interacting factors, including illness severity, perioperative instability, nutrition, and prolonged intensive care exposure.

## 8. Limitations of the Study

Evidence is heterogeneous by lesion type, severity, antibiotics, feeding practices, ICU course, sampling timepoints and analytic methods, limiting causal inference and cross-study comparability. Many data are observational, NEC is relatively uncommon (event-rate limitations), and probiotic/synbiotic studies differ by strains, dosing, timing, and endpoints. Safety estimates are weakened by inconsistent strain tracking and surveillance for probiotic bloodstream infection.

In addition, some mechanistic concepts discussed in the perioperative section rely on evidence from adult cardiac surgery populations, which may not be fully generalizable to neonates and young infants with congenital heart disease because of important developmental differences in gut barrier function, microbiome ecology, and immune responses.

As a narrative review, this article also has important methodological limitations. We did not perform a systematic search strategy, apply predefined study selection criteria, conduct a formal risk-of-bias assessment, or grade the certainty of evidence using a structured framework. Therefore, the synthesis should be interpreted as an integrative and hypothesis-generating overview rather than a formal systematic review or meta-analysis. These limitations restrict the ability to compare studies quantitatively, rank the strength of evidence across interventions, or draw definitive causal conclusions.

## 9. Future Research Perspectives

Prospective stage-based cohorts (pre-op → post-CPB → discharge → follow-up) should pair microbiome profiling with barrier injury markers, endotoxin activity, inflammatory mediators, and perfusion proxies. NEC studies should be lesion- and hemodynamics-stratified (ductal dependence, Doppler patterns, perfusion monitoring) to refine feeding protocols. Trials should define the target outcome (NEC, feeding tolerance, infection reduction, growth, Fontan inflammation) and include standardized safety monitoring for biotics. Also, future longitudinal studies should examine microbiome profiles together with growth and neurodevelopmental outcomes in order to clarify whether the microbiome is a mediator, a modifier, or simply a correlate of the phenotypes mentioned.

## 10. Conclusions

Congenital heart disease appears to create a state of baseline gut vulnerability that may be amplified by perioperative exposures, particularly cardiopulmonary bypass. The most consistently supported findings relate to perioperative intestinal barrier injury, endotoxemia, and inflammatory activation, as well as the clinical relevance of human milk exposure as a practical nutrition-based strategy in high-risk infants with complex congenital heart disease. These findings support the concept that the gastrointestinal tract should be considered a vulnerable target organ in congenital heart disease, especially during the neonatal and perioperative periods.

Other areas remain promising but less definitive. Synbiotics have demonstrated outcome benefits in a randomized trial of infants with cyanotic congenital heart disease, while probiotics may correct postoperative dysbiosis without consistently preventing intestinal injury. Similarly, Fontan circulation represents an emerging chronic phenotype in which dysbiosis, systemic inflammation, and protein-losing enteropathy may interact, but causality and therapeutic implications remain insufficiently established.

More speculative directions require further validation before clinical implementation. These include fecal microbiota transplantation, bile-acid modulation in Fontan physiology, and the potential relationship between early-life microbiome patterns, growth, and neurodevelopmental outcomes. Future studies should use longitudinal, stage-based designs that integrate microbiome profiling with intestinal barrier markers, endotoxin activity, inflammatory measures, perfusion indicators, and clinically meaningful outcomes. Such studies are needed to determine whether microbiome alterations are causal mediators, modifiable risk factors, or biomarkers of disease severity in children with congenital heart disease.

## Figures and Tables

**Table 1:** Key intervention-related studies and safety signals in the congenital heart disease gut–microbiome axis.

Authors	Population	Intervention or Exposure	Comparator	Outcomes Assessed	Main Findings
Cognata et al.	Neonates with complex congenital heart disease	Preoperative exclusive human milk feeding	Non-exclusive human milk or formula exposure	Preoperative necrotizing enterocolitis risk	Exclusive human milk feeding was associated with lower preoperative necrotizing enterocolitis risk, while higher feeding volumes and cow’s-milk formula exposure were linked to increased risk [30].
Zyblewski et al.	Neonates requiring cardiac surgery	Preoperative feeding strategy	Alternative feeding approach in randomized design	Intestinal barrier function	The study evaluated whether preoperative feeding affects intestinal barrier function in neonates requiring cardiac surgery [37].
Chen et al.	Infants after cardiac surgery	Early high-energy feeding	Standard feeding strategy	Growth, cardiac intensive care stay, ventilation time, infection rates	Early high-energy feeding was associated with improved growth and shorter cardiac intensive care stay, with reductions in ventilator time and postoperative infection rates [60].
Maki et al.	Infants after congenital heart surgery	Achievement of acute postoperative enteral nutrition targets	Not achieving predefined nutrition targets	Clinical outcomes and gastrointestinal complications	Achieving postoperative enteral nutrition targets was associated with improved clinical outcomes without necessarily increasing gastrointestinal complications [59].
Dilli et al.	Infants with cyanotic congenital heart disease	Enteral synbiotics	Placebo	Nosocomial sepsis, necrotizing enterocolitis, death	Synbiotics were associated with reduced nosocomial sepsis, necrotizing enterocolitis, and death compared with placebo [65].
Toritsuka et al.	Children undergoing cardiopulmonary bypass	Probiotics	Control group	Dysbiosis and intestinal injury endpoints	Probiotics corrected postoperative dysbiosis after cardiopulmonary bypass but did not sufficiently reduce intestinal injury [67].
Kocjancic et al.	Neonates with ductal-dependent congenital heart disease	Dual-strain probiotic	No probiotic exposure	Necrotizing enterocolitis	A possible reduction in necrotizing enterocolitis was suggested in a subgroup, but the overall cohort effect was not statistically definitive [68].
Wang et al.	Children after cardiac surgery	Probiotic exposure or ward-level probiotic use	Not applicable	Probiotic-associated bacteremia	Probiotic-associated bacteremia was reported after pediatric cardiac surgery, emphasizing the need for careful patient selection and infection-control measures [66].
Shah et al.	Adults with Fontan circulation	Colesevelam for bile-acid reduction	Crossover comparison in trial design	Bile-acid modulation in Fontan physiology	This trial design illustrates an emerging metabolite-directed strategy for Fontan physiology, although efficacy data remain early [69].

## Data Availability

No new data were created or analyzed in this study. Data sharing is not applicable to this article.
